# Efficacy of treatment with N‐acetylcysteine inhalation for AECOPD: A propensity‐score‐matched cohort study

**DOI:** 10.1111/crj.13690

**Published:** 2023-08-24

**Authors:** Hengyi Chen, Hui Zhou, Chen Luo, Kaican Zong, Yingya Fu, Wen Li, Chunyan Luo, Guojuan Xue, E. Jiang, Yang Duan, Tinglan Luo, Yangzhi Jiang

**Affiliations:** ^1^ Department of Pulmonary and Critical Care Medicine the Seventh People's Hospital of Chongqing Chongqing China

**Keywords:** AECOPD, clinical efficacy, hospitalization, N‐acetylcysteine inhalation

## Abstract

**Introduction:**

N‐acetylcysteine (NAC) prevents acute exacerbations of chronic obstructive pulmonary disease (AECOPD). However, the value of NAC inhalation in the treatment of patients with AECOPD is still poorly understood. The study was conducted to evaluate the efficacy of NAC inhalation in AECOPD patients requiring hospitalization.

**Methods:**

In this single institutional, retrospective cohort study, all patients with AECOPD requiring hospitalization between January 2021 and January 2022 were included. Patients were divided into NAC group and Non‐NAC group according to whether being treated with NAC inhalation and were matched using the propensity score. The primary outcome was a composite of progression to ventilation requirement, in‐hospital mortality and readmission for AECOPD within 30 days. The effect on the mean hospitalized days, blood gas indexes and the incidence rate of adverse drug events were compared between the two groups.

**Results:**

Ninety‐six patients in the NAC group were matched with 96 patients in the Non‐NAC group. The differences in the primary composite end point (NAC group vs Non‐NAC group, 5.2% vs 16.7%; *P* = 0.011) were significant. The median time to discharge was shorter in the NAC group (8.3 vs. 9.1 days, *P* = 0.030). The NAC group presented a larger increase in partial pressure of arterial oxygen (P_a_O_2_) and a higher ratio of self‐reported symptomatic improvement from admission to day 5. There was no definite difference between the two groups in the frequency of adverse event.

**Conclusion:**

NAC inhalation is associated with an improved clinical outcome. A further study should be conducted to confirm the clinical usefulness of NAC inhalation in AECOPD patients.

## Graphical abstract

This cohort study showed that NAC inhalation is associated with an improved clinical outcome for patients with AECOPD, including reducing machine ventilation rate, shortening hospitalization days, improving patient symptoms and raising P_a_O_2_, without increasing toxicity. Further studies are needed to confirm the results. The application of NAC inhalation in AECOPD was worth further study.

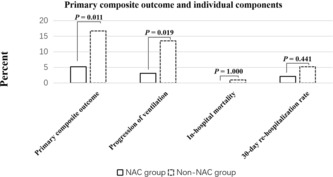

**Abbreviations**
AECOPD: acute exacerbations of chronic obstructive pulmonary disease
BMI: body mass index
CAT: COPD assessment test
COPD: chronic obstructive pulmonary disease
FEF_25–75%_
: forced expiratory flow between 25% and 75% of vital capacity
FEF_50%_
: forced expired flow at 50% of vital capacity
FEV_1_
: forced expiratory volume in 1 s
FVC: forced vital capacity
GOLD: global initiative for chronic obstructive lung disease
NAC: N‐acetylcysteine
P_a_CO_2_
: partial pressure of carbon dioxide
P_a_O_2_
: partial arterial oxygen pressure
PSM: propensity score match
RV/TLC: residual volume/total lung capacity
RV/TLC: residual volume/total lung capacity

## 1 BACKGROUND

Chronic obstructive pulmonary disease (COPD) is one of the most prevalent chronic pulmonary diseases and a major public health issue all over the word.^1^ Acute exacerbations of COPD (AECOPD) are the important cause of emergency hospitalization,^2^ causing a substantial social‐economic burden with significant morbidity and mortality.^3^ Therapeutically, AECOPD are approached as a single disease entity and treated routinely with antibiotics, bronchodilators, expectorants and systemic corticosteroids,^4^ but optimization management of AECOPD represents an important clinical challenge.^5^

A series of studies have shown that increased oxidative stress is not only the most important pathogenic mechanisms of COPD,^6^ but also one of the important factors leading to AECOPD.^7^ Biomarkers associated with oxidative stress such as serum gamma‐glutamyltransferase,^8^ 8‐Hydroxy‐2′‐ deoxyguanosine,^9^ superoxide dismutase and malondialdehyde levels^10^ were evidently increased in AECOPD patients compared with that inpatients with stable COPD and healthy persons. The abnormally elevated oxidative stress can weaken macrophage function, thus resulting in impaired responses towards exacerbating triggers and contributing to excessive inflammation of the airways.^11^ Therapeutic intervention targeting the oxidative stress may give the possibility to improve the prognosis of patients with AECOPD.

N‐acetylcysteine (NAC), a precursor of glutathione and a synthetic derivative of the endogenous amino acid L‐cysteine, has been proposed not only as an expectorant but also as a therapeutic agent in a variety of disorders involving oxidative stress.^12^ For the past several decades, it is widely used in several lung disorders as a mucolytic.^13^

Although NAC has been shown to prevent the occurrence of AECOPD,^14^ as well as the pre‐clinical data about the use of NAC inhalation in AECOPD indicate a positive efficacy, its therapeutic value in the treatment of AECOPD has yet to be explored in further studies. Accordingly, the present research was to evaluate the clinical efficacy of NAC inhalation in treating patients with AECOPD requiring hospitalization.

## 2 METHODS

### 2.1 Study design and patients

In this single‐centre, retrospective cohort study, patients (≥50 years) diagnosed with AECOPD requiring hospitalization referred to the Pulmonary and Critical Care Medicine Unit of Seventh People's Hospital of Chongqing between January 2021 and January 2022 were investigated. The diagnosis of COPD was supported by spirometric evidence of airflow obstruction even with bronchodilator (forced expiratory volume in 1 s/forced vital capacity < 0.70) when clinically stable.^15^ AECOPD were defined as an acute worsening of respiratory symptoms (cough, sputum, breathlessness) that lead to the need for additional treatment.^15^ The COPD ‘frequent exacerbator’ phenotype was defined by at least two moderate‐to‐severe exacerbations per year.^16^ All the included patients had a minimum of two arterial blood gas measurements and pulmonary function tests. The test time were between 24 h before admission and 24 h after admission, and the time between each two tests should be no less than 5 days.

Exclusion criteria were the presence of multidrug‐resistant bacteria infection, invasive fungal infection, pulmonary embolism, asthma, allergic rhinitis or atopy, interstitial lung disease, lung cancer, bronchiectasis or active pulmonary tuberculosis, acute heart failure, cerebrovascular accident, acute respiratory acidosis needing non‐invasive ventilation, severe heart, liver, kidney, endocrine diseases or gastrointestinal tract disease. Patients were also excluded from the study if the length of NAC inhalation therapy was less than 5 days or had been exposed to NAC by oral administration during hospitalization.

**FIGURE 1:**
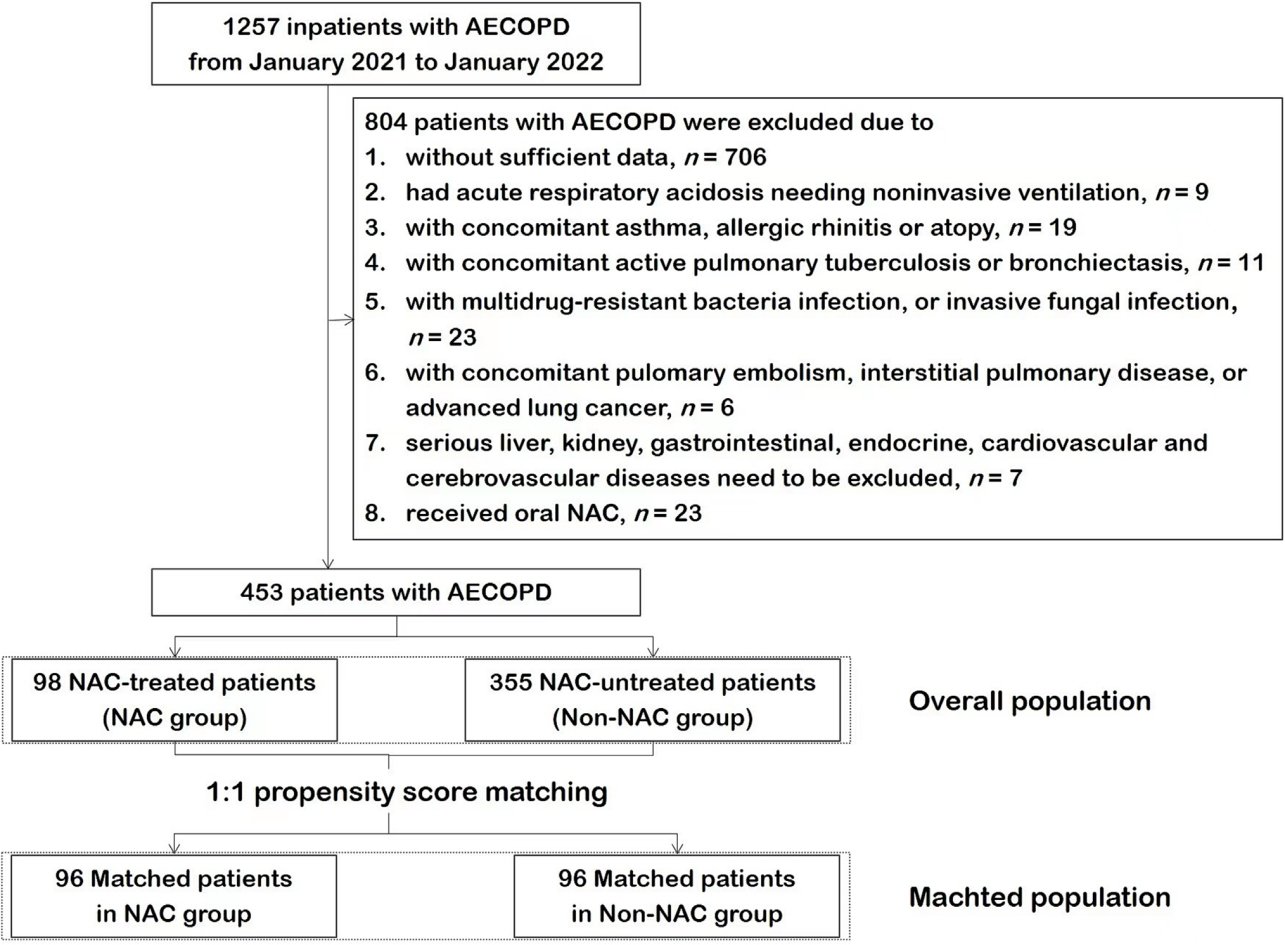
Flow diagram illustrating propensity score matching in patients with AECOPD. AECOPD, acute exacerbations of chronic obstructive pulmonary disease; NAC, N‐acetylcysteine.

A total of 453 patients with AECOPD were divided into two groups: patients who received aerosol inhalation of 3‐mL NAC (10%) (Tandi, Hunan Warrant Pharmaceutical CO., LTD, China; No. H20183186) twice daily for 5–10 days while receiving conventional therapy, including oxygen therapy, bronchodilators, systemic glucocorticoids, antibiotics and expectorants were defined as the NAC group, and those treated just with conventional therapy were defined as the Non‐NAC group. The patient recruitment flow diagram is presented in Figure 1.

### 2.2 Outcomes definitions

The primary outcome was a composite of progression to ventilation requirement, in‐hospital mortality and 30‐day re‐hospitalization rate. Secondary outcomes included the length of hospital stay, the changes in patient‐reported symptoms during the course of hospital admission, partial pressure of arterial oxygen (P_a_O_2_), arterial partial pressure of carbon dioxide (P_a_CO_2_) and the dosage of systemic glucocorticoids. The incidence of adverse drug events, including decreased appetite, nausea, constipation, rash, stomatitis and urticarial, were calculated to estimate the differences between the two groups.

### 2.3 Statistical analysis

All quantitative data were presented as mean ± SD. Discrete variables were compared using the chi‐squared test. The changes in continuous variables between and in the two groups were compared using ANOVA and paired *t*‐tests according to the distribution of the data. Continuous variables were logarithmically transformed for further analysis in case of skewed distributions. Stratified analysis was performed to analyse the efficacy of NAC inhalation in patients with or without type II respiratory failure. The Kaplan–Meier method and log‐rank test were used to analyse the effect of NAC inhalation on the length of hospital stay. According to guidelines,^17^ propensity score matching (PSM) was strictly performed to match patients with AECOPD between the two groups and was calculated for each patient with a multivariate logistic regression model according to baseline characteristics. The following variables were involved in the model: sex, age, current smoking status, time of exacerbation, COPD assessment test (CAT) score, type II respiratory failure, COPD stage, ‘Frequent Exacerbator’ phenotype, concomitant medication use, P_a_O_2_ and P_a_CO_2_.

The PSM was implemented by using the 1:1 nearest‐neighbour method with a 0.1 calliper width. The matching quality was assessed by comparing the two groups before and after PSM to define whether a balance was achieved after matching.

Statistical analyses were carried out using SPSS software, version 26.0 (SPSS, Inc., Chicago, IL, US). A *P*‐value of <0.05 was considered statistically significant.

## 3 RESULTS

### 3.1 Baseline characteristics

In total, 453 patients were included in the analysis, all of whom were Asian, with 98 in the NAC group and 355 in the Non‐NAC group (Figure 1). As shown in Table 1, the two groups were similar in most baseline parameters, including age, sex, body mass index (BMI), current smoking status, P_a_O_2_, type II respiratory failure, pulmonary function, COPD stage, ‘Frequent Exacerbator’ phenotype, systemic corticosteroids utilization ratio and comorbidities. In comparison with the Non‐NAC group, patients in the NAC group had longer time of exacerbation (*P* = 0.041), higher CAT score (*P* = 0.000) and P_a_CO_2_ (*P* = 0.010). In addition, the using rates of inhaled corticosteroids, theophylline and expectorant were significantly lower in the NAC group than in the Non‐NAC group.

**TABLE 1:** Baseline characteristics of hospitalized AECOPD patients before propensity score matching.

Characteristics	Overall population		
	NAC group (*n* = 98)	Non‐NAC group (*n* = 355)	*P*‐value
Age (years)	70.7 ± 8.14	69.2 ± 8.03	0.110
Sex			
Male	75 (76.5%)	280 (78.9%)	0.618
Female	23 (23.5%)	75 (21.1%)	
BMI (kg/m^2^)	22.0 ± 1.35	22.2 ± 1.43	0.185
Current smokers	21 (21.4%)	69 (19.4%)	0.662
Time of exacerbation (day)	7.0 ± 3.00	6.3 ± 2.63	0.041
CAT score	22.4 ± 3.87	20.3 ± 4.42	0.000
P_a_O_2_ mmHg	68.6 ± 8.41	69.9 ± 10.33	0.250
P_a_CO_2_ mmHg	49.4 ± 9.87	46.9 ± 7.90	0.010
PH	7.4 ± 0.04	7.4 ± 0.04	0.155
Type II respiratory failure	38 (38.8%)	103 (29.0%)	0.065
Baseline spirometry			
FEV_1_%pred	46.1 ± 17.50	46.8 ± 17.91	0.750
FVC%pred	69.9 ± 18.08	71.8 ± 16.92	0.311
FEV_1_/FVC (%)	51.9 ± 9.48	50.6 ± 11.23	0.289
FEF_50%_pred	16.5 ± 9.32	17.0 ± 8.74	0.662
FEF_25–75%_pred	15.6 ± 8.07	16.0 ± 7.42	0.717
RV/TLC	54.5 ± 6.80	54.1 ± 6.49	0.598
GOLD stage			
1	4 (4.1%)	12 (3.4%)	0.095
2	27 (27.6%)	142 (40.0%)	
3	48 (49.0%)	130 (36.6%)	
4	19 (19.4%)	71 (20.0%)	
‘Frequent Exacerbator’ phenotype	61 (62.2%)	249 (70.1)	0.137
Concomitant medication use, No. (%)			
Systemic corticosteroids	93 (94.9%)	341 (96.1%)	0.613
Inhaled corticosteroids	81 (82.7%)	325 (91.6%)	0.011
Inhaled bronchodilator	94 (95.9%)	349 (98.3%)	0.154
Theophylline	95 (96.9%)	353 (99.4%)	0.036
Expectorant	74 (75.5%)	341 (96.1%)	0.000
Antibiotic	92 (93.9%)	336 (94.7%)	0.768
Comorbidities			
Hypertension	31 (31.3%)	84 (23.7%)	0.109
Type 2 diabetes mellitus	5 (5.1%)	18 (5.1%)	0.990
Ischaemic heart disease	37 (37.8%)	102 (28.7%)	0.086
Pulmonary heart disease	18 (18.4%)	72 (20.3%)	0.674

Abbreviations: BMI, body mass index; CAT, COPD assessment test; P_a_O_2_, partial arterial oxygen pressure; P_a_CO_2_, partial pressure of carbon dioxide; FEV_1_, forced expiratory volume in 1 s; FVC, forced vital capacity; FEF_50%_, forced expired flow at 50% of vital capacity; FEF_25–75%_, forced expiratory flow between 25% and 75% of vital capacity; RV/TLC, residual volume/total lung capacity; GOLD, global initiative for chronic obstructive lung disease.

We then performed a 1:1 PSM analysis to minimize selection bias. This procedure resulted in a matched cohort, including 96 patients in the NAC group and 96 patients in the Non‐NAC group (Table 2). There was no statistical difference in clinical features between the two groups.

**TABLE 2:** Baseline characteristics of hospitalized AECOPD patients in NAC and non‐NAC groups matched by propensity score.

Characteristics	Matched population			
	NAC group (*n* = 96)	Non‐NAC group (*n* = 96)	*P*‐value	SMD
Age (years), mean ± SD	70.8 ± 8.13	71.8 ± 7.13	0.386	−0.09
Sex, No. (%)				
Male	73 (76.0%)	70 (72.9%)	0.619	‐
Female	23 (24.0%)	26 (27.1%)		
BMI (kg/m^2^), mean ± SD	22.0 ± 1.34	21.8 ± 1.52	0.363	0.05
Current smokers, No. (%)	20 (20.8%)	21 (21.9%)	0.860	‐
Time of exacerbation (day), mean ± SD	7.0 ± 2.98	6.6 ± 2.76	0.316	0.06
CAT score, mean ± SD	22.6 ± 3.74	22.5 ± 4.44	0.833	0.01
P_a_O_2_ mmHg, mean ± SD	68.7 ± 8.31	66.9 ± 9.56	0.183	0.06
P_a_CO_2_ mmHg, mean ± SD	49.6 ± 9.87	50.0 ± 9.98	0.738	−0.14
PH, mean ± SD	7.4 ± 0.04	7.4 ± 0.05	0.553	−0.02
Type II respiratory failure	38 (39.6%)	40 (41.7%)	0.769	‐
Baseline spirometry, mean ± SD				
FEV_1_%pred	46.1 ± 17.63	45.3 ± 19.11	0.743	0.01
FVC%pred	70.0 ± 18.19	69.1 ± 18.30	0.746	0.03
FEV_1_/FVC(%)	51.8 ± 9.55	49.7 ± 11.83	0.177	0.15
FEF_50%_pred	16.6 ± 9.41	15.5 ± 9.50	0.428	0.02
FEF_25–75%_pred	15.7 ± 8.15	14.9 ± 7.86	0.478	0.06
RV/TLC	54.6 ± 6.81	56.5 ± 7.71	0.074	−0.14
GOLD stage				
1	4 (4.2%)	5 (5.2%)	0.419	‐
2	26 (27.1%)	29 (30.2%)		
3	47 (49.0%)	36 (37.5%)		
4	19 (19.8%)	26 (27.1%)		
‘Frequent Exacerbator’ phenotype	61 (63.5%)	64 (66.7%)	0.650	‐
Concomitant medication use, No. (%)				
Systemic corticosteroids	92 (95.8%)	90 (93.8%)	0.516	‐
Inhaled corticosteroids	81 (84.4%)	83 (86.5%)	0.683	‐
Inhaled bronchodilator	94 (97.9%)	94 (97.9%)	1.000	‐
Theophylline	95 (99.0%)	96 (100.0%)	1.000	‐
Expectorant	72 (75.0%)	78 (81.3%)	0.295	‐
Antibiotic	90 (93.8%)	93 (96.9%)	0.495	‐
Comorbidities, No. (%)				
Hypertension	30 (31.3%)	23 (24.0%)	0.258	‐
Type 2 diabetes mellitus	5 (5.2%)	9 (9.4%)	0.267	‐
Ischaemic heart disease	36 (37.5%)	30 (31.3%)	0.362	‐
Pulmonary heart disease	16 (16.7%)	25 (26.0%)	0.113	‐

*Note*: SMD, standardized mean difference for continuous variables with normal distribution. Values up to +0.2 or −0.2 denote a small effect.

Abbreviations: BMI, body mass index; CAT, COPD assessment test; P_a_O_2_, partial arterial oxygen pressure; P_a_CO_2_, partial pressure of carbon dioxide; FEV_1_, forced expiratory volume in 1 s; FVC, forced vital capacity; FEF_50%_, forced expired flow at 50% of vital capacity; FEF_25–75%_, forced expiratory flow between 25% and 75% of vital capacity; RV/TLC, residual volume/total lung capacity; GOLD, global initiative for chronic obstructive lung disease.

### 3.2 Therapeutic effect on clinical outcomes

As shown in Table 3, the prevalence of the primary end point of the NAC group was 5.2% (*n* = 5), which was significantly lower than that of the Non‐NAC group (*n* = 16, 16.7%; *P* = 0.011). The rate of progression to need for ventilation was 3.1% (*n* = 3) in the NAC group and was lower than the rate of 13.5% (*n* = 13) in the Non‐NAC group; the difference had statistical significance (*P* = 0.019). One death occurred in the Non‐NAC group, and the cause of death was cardiovascular incidence. There were no significant differences in the in‐hospital mortality (0 vs. 1.0%; *P* = 1.000) and 30‐day re‐hospitalization rate (2.1% vs. 5.2%; *P* = 0.441) between the two matched groups. Furthermore, a benefit of NAC inhalation on reducing machine ventilation rate was observed in patient with type II respiratory failure (OR = 0.23, 95% CI = 0.06–0.89), but no statistical significance was observed in patients without type II respiratory failure in advanced analysis (Table 4).

**TABLE 3:** Primary composite outcome and individual components of hospitalized AECOPD patients after propensity score matching.

	NAC group (*n* = 96)	Non‐NAC group (*n* = 96)	*P*‐value
Primary composite outcome, No. (%)	5 (5.2%)	16 (16.7%)	0.011
Progression of ventilation, No. (%)	3 (3.1%)	13 (13.5%)	0.019
In‐hospital mortality, No. (%)	0	1 (1.0%)	1.000
30‐day re‐hospitalization rate, No. (%)	2 (2.1%)	5 (5.2%)	0.441

**TABLE 4:** Effects of NAC inhalation on progression of ventilation in patients with or without type II respiratory failure.

Type II respiratory failure	Progression of ventilation				*χ* ^2^	*P*‐value	Odds ratio (95% CI)
	Yes		NO				
	NAC	Non‐NAC	NAC	Non‐NAC			
Yes	3 (3.1%)	11 (11.5%)	35 (36.5%)	29 (30.2%)	5.086	0.024	0.23 (0.06–0.89)
No	0	2 (2.1%)	58 (60.4%)	54 (56.3%)	2.108	0.146	0.48 (0.40–0.58)
Total	3 (3.1%)	13 (13.5%)	93 (96.9%)	83 (86.5%)	6.818	0.009	0.21 (0.06–0.75)

**FIGURE 2:**
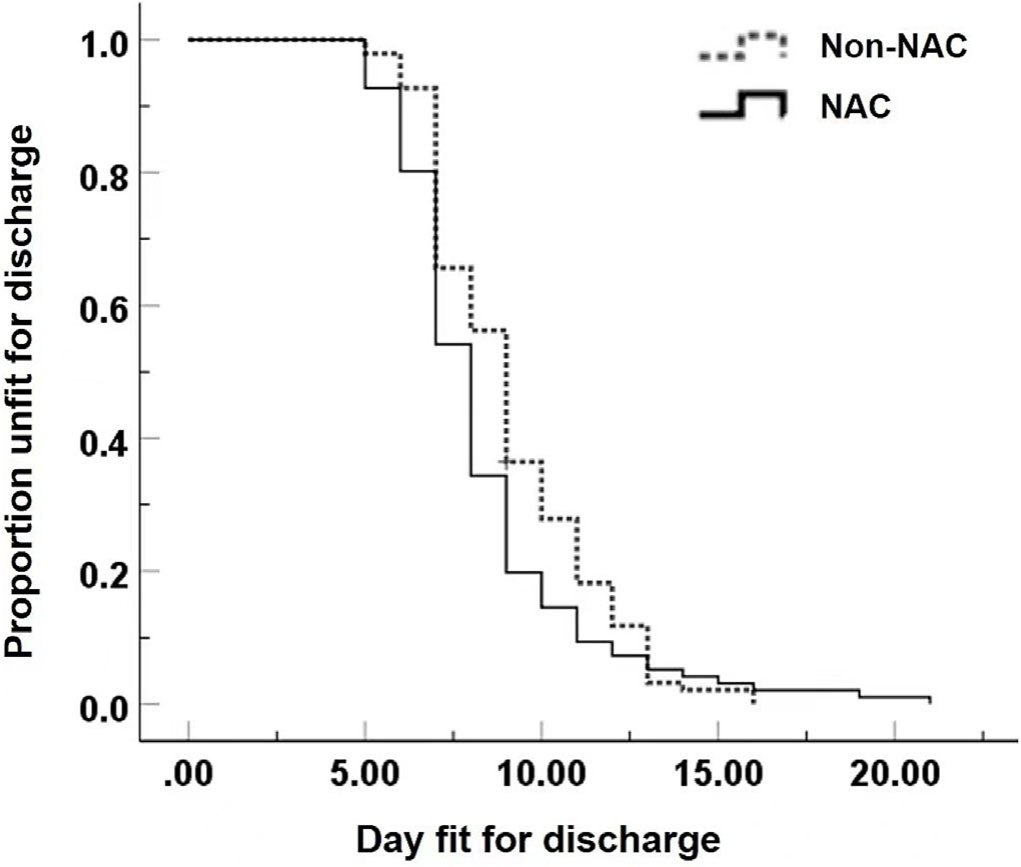
Kaplan–Meier plot of the proportion of AECOPD patients remaining in hospital after propensity score matching.

In addition, the NAC group was correlated with the shorter length of hospital stay compared with the Non‐NAC groups (Table 5). The median length of hospital stay was 8.3 days in the NAC group (95% CI, 7.6 to 8.4 days) and 9.1 days in the Non‐NAC group (95% CI, 8.5 to 9.5 days; *P* = 0.030; Figure 2). Regarding the influence of predictive factors on the length of hospital stay, univariate Cox regression analysis showed that various variables, including type II respiratory failure, GOLD stage, ‘Frequent Exacerbator’ phenotype, systemic corticosteroids use, NAC inhalation, ischaemic heart disease and pulmonary heart disease, were obviously associated with hospital stay (Table S1). Multivariate analyses showed that GOLD stage 1 or 2, ‘Frequent Exacerbator’ phenotype, systemic corticosteroids use and NAC inhalation were associated with shortened hospital stay, whereas GOLD Stage 3 or 4 was related with prolonged hospitalization (Table S2).

**TABLE 5:** Clinical outcomes of hospitalized AECOPD patients after propensity score matching.

	NAC group (*n* = 96)	Non‐NAC group (*n* = 96)	*P*‐value
Length of hospital stay (days), mean ± SD	8.3 ± 2.76	9.1 ± 2.38	0.037
Self‐reported symptomatic improvement from admission to day 5, No. (%)	82 (85.4%)	70 (72.9%)	0.033
Actual total dose of prednisone (mg), mean ± SD	304.6 ± 173.2	315.5 ± 141.3	0.633

Meanwhile, self‐reported symptomatic improvement from admission to day 5 in the NAC group was 85.4% and which was statistically higher than that in the Non‐NAC group (72.9%; *P* = 0.033). Though the average total dose of prednisone equivalent used in the NAC group was slightly lower than that in the Non‐NAC group, no statistical difference was shown between the two groups (Table 5).

Compared with the level before treatment, P_a_O_2_ in the NAC group and the Non‐NAC group improved prominently after treatment, and the improvement in the NAC group was significantly higher than that in the Non‐NAC group (*P* < 0.05). Moreover, the mean P_a_CO_2_ were obviously improved in all AECOPD patients, and there was no significant difference between the two groups (Table S3).

### 3.3 Safety

The incidence of adverse drug events was similar between groups (Table 6). All adverse drug events were mild and did not require dose reductions or discontinuation of therapy. Drug‐related adverse reactions in two groups included decreased appetite, nausea, constipation, rash, stomatitis and urticaria; the incidences of such reactions showed no difference between the NAC (41.7%) and Non‐NAC (40.6%) groups (*P* = 0.883), and no severe adverse reactions were related to NAC inhalation. These results indicated that the addition of inhaled NAC did not substantially change the tolerance to treatment in patients with AECOPD.

**TABLE 6:** Summary of adverse drug events.

	NAC group (*n* = 96)	Non‐NAC group (*n* = 96)	*P*‐value
Treatment‐related adverse events, No. (%)	40 (41.7%)	39 (40.6%)	0.883
Decreased appetite	12 (12.5%)	11 (11.5%)	0.824
Nausea	8 (8.3%)	5 (5.2%)	0.389
Constipation	9 (9.4%)	10 (10.4%)	0.809
Rash	4 (4.2%)	4 (4.2%)	0.718
Stomatitis	5 (5.2%)	6 (6.3%)	0.756
Urticaria	2 (2.1%)	3 (3.1%)	1.000

## 4 DISCUSSION

The results of this retrospective cohort study indicate that NAC inhalation in combination with routine treatment was associated with a reduced risk for treatment failure, as measured by the composite end point of progression to ventilation requirement, in‐hospital mortality and readmission within 30 days in Asian patients with AECOPD compared with a routine medical treatment, which is mainly benefited from the effect of NAC inhalation in reducing machine ventilation rate of AECOPD patients with type II respiratory failure. Meanwhile, NAC inhalation was associated with shorter hospital stays and might play a role in improving patient symptoms and raising P_a_O_2_ without increasing toxicity.

COPD is one of the most common chronic respiratory diseases that have brought great economic and social burdens for both sufferers and health care systems.^18^ In particular, nearly 35% to 84% of the COPD medical costs are due to AECOPD.^19^ It has been reported that taking orally high‐dose NAC markedly improved pulmonary function in COPD patients on stable phase.^20^ Also, it has been suggested that patients with AECOPD can effectively diminish their symptoms of cough, sputum and breathing difficulty after taking NAC.^21^, ^22^ However, there is little study concerning the clinical effects of NAC inhalation in patients with AECOPD. Inhalation therapy is the primary way for AECOPD in clinical practice, and about 78.2% of AECOPD patients used a combination regimen that mainly involve a ICS, and either a long‐acting or a short‐acting β‐receptor agonist.^19^ This report demonstrated the potential benefit of NAC inhalation on improving the treatment effect of AECOPD requiring hospitalization. In clinical, it would be an effective supplementary treatment to patients with AECOPD.

Patients with AECOPD suffer from worsening of clinical parameters, which are often associated with systemic inflammatory response and increasing hypercapnia.^23^ As one of the common complications of AECOPD, acute respiratory failure is reported as increasing the risk of mortality of patients, particularly type II respiratory failure.^24^ Prompt correction of acute respiratory failure plays an important role in improving the prognosis of AECOPD. Mechanical ventilation can be successfully used for patients with acute hypercapnic breathing failure caused by AECOPD but accompanied by high costs and long hospital stays.^25^ So, some researchers begin to explore the probability of high‐flow nasal cannula substituting the non‐invasive ventilation in such patients.^26^ Our result showed that about 40% of patients suffered from type II respiratory failure, and there was no statistical significance in the incidence of type II respiratory failure between the NAC and Non‐NAC groups. Notably, NAC inhalation was related to the lower rate of machine ventilation. Further, the results of this study did find that the rate of self‐reported symptomatic improvement from admission to day 5, and the improved degree of P_a_O_2_ in the NAC group was higher than that in the Non‐NAC group. As all individuals in this research received adequate therapy including symptomatic treatment, the benefit of NAC inhalation might not only stem from the expectorant action of NAC but also stem from regulation of oxidative stress and inhibition of airway inflammation. Mucus hypersecretion is a distinguishing pathophysiological feature of COPD.^27^ As an expectorant, NAC can reduce sputum viscosity, as well as contribute to the improvement of symptoms and pulmonary function by breaking the disulfide bonds of heavily cross‐linked mucins.^12^ What is more, it was reported that pro‐inflammatory cytokines as well as makers of oxidative stress are noticeably over‐expressed in acute aggressive periods compared with those in stable period for COPD patients, which are inversely associated with FEV_1_ and FEV_1_/FVC, and seem to be positively correlated to increased airway inflammation and bronchial structure remodeling^28^; the function of NAC inhalation to modulate oxidative stress benefits the improvement of the pulmonary function. NAC can modulate not only oxidative stress but also other pathophysiologic procedure including inflammation, apoptosis and the dysfunction of mitochondria^12^ so as to ameliorate pulmonary function and improve curative effect of AECOPD patients.

Compared with intravenous or oral medication, inhalation administration is delivered by a specific inhalation device by which that can exert a direct and rapid effect on the trachea and bronchus to achieve high local drug concentrations with reducing systemic influence. Inhalant NAC solutions have good compatibility with other agents^29^ and so are likely to be safe and well‐tolerated. Indeed, this research did not find additional adverse drug effects with NAC inhalation. The results indicated that NAC inhalation was well‐tolerated in AECOPD patients.

The present research also has limitations. This retrospective study was conducted in a single unit with a short follow‐up period. Further multi‐centre, prospective clinical research with a larger sample of patients and longer follow‐up periods are needed to evaluate the clinical efficacy of NAC inhalation more clearly in patients with AECOPD, as well as to explore the mechanism of action.

## 5 CONCLUSIONS

In conclusion, NAC inhalation in conjunction with routine treatment is associated with lower machine ventilation and re‐hospitalization rates in patients with AECOPD but did not result in differences in in‐hospital mortality compared with routine treatment. Because of the limitations of this study, further multi‐centre, prospective studies are needed to confirm the results.

## AUTHOR CONTRIBUTIONS

Hengyi Chen, Hui Zhou, Chen Luo and Kaican Zong contributed to the study conception and design. Chunyan Luo, Yang Duan and Tinglan Luo acquired the data. Hengyi Chen, Kaican Zong, Yingya Fu, Wen Li and Guojuan Xue analysed and interpreted the clinical data. Yangzhi Jiang is responsible for data quality control. Hengyi Chen and Kaican Zong drafted the manuscript. All authors read and approved the final manuscript.

## CONFLICT OF INTEREST STATEMENT

There are no conflicts of interest.

## ETHICS APPROVAL AND CONSENT TO PARTICIPATE

The study was approved by the Ethical Committee of the Seventh People's Hospital of Chongqing (No.2021017). The study is conducted according to the Declaration of Helsinki. Informed consent to participate is not applicable in this retrospective study and is waived by the Ethical Committee of the Seventh People's Hospital of Chongqing.

## CONSENT FOR PUBLICATION

Not applicable.

## Supporting information


**Table S1.** Univariate analyses of prognostic factors for hospital stay in patients with AECOPD. BMI, Body Mass Index; CAT, COPD assessment test; GOLD, global initiative forchronic obstructive lung disease.
**Table S2.** Multivariate analyses of prognostic factors for prognostic factors for hospital stay in patients with AECOPD. GOLD, global initiative forchronic obstructive lung disease.
**Table S3.** Changes arterial blood gas tensions breathing air in hospitalized AECOPD during the course of hospital admission.
PaO2, partial arterial oxygen pressure; PaCO2, partial pressure of carbon dioxide. *p < 0.05 compared to baseline; #*p* < 0.05 compared to Non‐NAC group.

## Data Availability

The data that support the findings of this study are available from the corresponding author upon reasonable request.
