# Supplementary material for: Efficacy of treatment with N‐acetylcysteine inhalation for AECOPD: A propensity‐score‐matched cohort study
Source: Clin Respir J. 2023 Aug 24;17(10):1038–47. doi: 10.1111/crj.13690 (PMC10543066; doi:10.1111/crj.13690)
Supplement: Supplementary file 1 — Table S1. Univariate analyses of prognostic factors for hospital stay in patients with AECOPD. BMI, Body Mass Index; CAT, COPD assessment test; GOLD, global initiative forchronic obstructive lung disease. Table S2. Multivariate analyses of prognostic factors for prognostic factors for hospital stay in patients with AECOPD. GOLD, global initiative forchronic obstructive lung disease. Table S3. Changes arterial blood gas tensions breathing air in hospitalized AECOPD during the course of hospital admission. PaO2, partial arterial oxygen pressure; PaCO2, partial pressure of carbon dioxide. *p < 0.05 compared to baseline; #p < 0.05 compared to Non‐NAC group. [file CRJ-17-1038-s001.docx]

**S1 Table** Univariate analyses of prognostic factors for hospital stay in patients with AECOPD.

| **Variable** | **Hospital stay** | |
| --- | --- | --- |
|  | **Hazard ratio (95% CI)** | ***P* value** |
| Age(yrs), mean±SD | 0.99(0.97-1.01) | 0.162 |
| BMI(kg/m^2^) | 1.05(0.95-1.16) | 0.341 |
| Current smokers | 1.04(0.73-1.49) | 0.814 |
| CAT score | 0.98(0.95-1.02) | 0.285 |
| Type Ⅱ Respiratory failure | 2.19(1.61-2.99) | <0.001 |
| GOLD stage |  | <0.001 |
| 1 | 0.46(0.06-0.75) |  |
| 2 | 0.94(0.14-0.99) |  |
| 3 | 1.45(1.22-9.45) |  |
| 4 | 3.22(1.48-12.31) |  |
| “Frequent Exacerbator” phenotype | 1.87(1.37-2.56) | <0.001 |
| Systemic corticosteroids | 0.35(0.18-0.69) | 0.003 |
| Inhaled corticosteroids | 0.86(0.58-1.29) | 0.473 |
| Inhaled NAC | 0.73(0.55-0.98) | 0.036 |
| Hypertension | 0.93(0.67-1.27) | 0.631 |
| Type 2 diabetes mellitus | 1.25(0.71-2.20) | 0.445 |
| Ischaemic heart disease | 1.46(1.07-1.98) | 0.016 |
| Pulmonary heart disease | 0.44(0.31-0.63) | <0.001 |

BMI, Body Mass Index; CAT, COPD assessment test; GOLD, global initiative forchronic obstructive lung disease.

**S2 Table** Multivariate analyses of prognostic factors for prognostic factors for hospital stay in patients with AECOPD.

| **Variable** | **Hospital stay** | |
| --- | --- | --- |
|  | **Hazard ratio (95% CI)** | ***P* value** |
| Type Ⅱ Respiratory failure | 1.21(0.82-1.77) | 0.335 |
| GOLD stage |  | <0.001 |
| 1 | 0.36(0.05-0.41) |  |
| 2 | 0.86(0.26-0.98) |  |
| 3 | 1.49(1.17-5.72) |  |
| 4 | 1.75(1.29-10.57) |  |
| “Frequent Exacerbator” phenotype | 1.55(1.12-2.14) | 0.008 |
| Systemic corticosteroids | 0.45(0.22-0.92) | 0.029 |
| Inhaled NAC | 0.72(0.53-0.97) | 0.030 |
| Ischaemic heart disease | 1.35(0.97-1.88) | 0.074 |
| Pulmonary heart disease | 0.67(0.43-1.04) | 0.075 |

GOLD, global initiative forchronic obstructive lung disease.

**S3 Table** Changes arterial blood gas tensions breathing air in hospitalized AECOPD during the course of hospital admission.

| **Characteristics** | **Group** | | | |
| --- | --- | --- | --- | --- |
|  | **NAC group**  **(n=96)** | | **Non-NAC group**  **(n=96)** | |
|  | Baseline | At 5-7 days | Baseline | At 5-7 days |
| P_a_O_2_ mmHg, mean±SD | 68.7±8.31 | 84.9±7.60*^＃^ | 66.9±9.56 | 81.0±9.29* |
| P_a_CO_2_ mmHg, mean±SD | 49.6±9.87 | 44.8±7.84* | 50.0±9.98 | 45.2±6.20* |

P_a_O_2_, partial arterial oxygen pressure; P_a_CO_2_, partial pressure of carbon dioxide. **p*<0.05 compared to baseline; ^＃^*p*<0.05 compared to Non-NAC group.
